# P75 neurotrophin receptor positively regulates the odontogenic/osteogenic differentiation of ectomesenchymal stem cells via nuclear factor kappa-B signaling pathway

**DOI:** 10.1080/21655979.2022.2063495

**Published:** 2022-04-29

**Authors:** Peifen Shan, Xiaole Wang, Yanyan Zhang, Zhisheng Teng, Yunxiao Zhang, Qiu Jin, Jiefan Liu, Jianfeng Ma, Xin Nie

**Affiliations:** a Department of Prosthodontics, School & Hospital of Stomatology, Wenzhou Medical University, Wenzhou, China; b Department of Nursing, School & Hospital of Stomatology, Wenzhou Medical University, Wenzhou, China; c Department of Oral and Maxillofacial Surgery, School & Hospital of Stomatology, Wenzhou Medical University, Wenzhou, China

**Keywords:** p75NTR MSCs NF-κB cell differentiation tooth development

## Abstract

p75NTR, a neural crest stem cell marker, is continuously expressed in mesenchymal cells during tooth development. Importantly, high expression of p75NTR in the late bell stage implicates its involvement in odontogenesis and mineralization. However, the regulatory mechanisms underlying p75NTR involvement in odonto/osteogenic differentiation remain unclear. Here, we investigate the effect and potential mechanisms underlying p75NTR involvement in odonto/osteogenic differentiation. We dissected EMSCs from the first branchial arches of mice embryo and compared the proliferation and migration of p75NTR^+/+^ and p75NTR^−/−^EMSCs by transwell, scratch and cell counting kit 8(CCK8)assays. The differentiation ability and the involvement of nuclear factor kappa-B (NF-κB) pathway were investigated through alkaline phosphatase and immunofluorescence assay, real-time PCR, and western blot. During induction of dental epithelium conditioned medium, p75NTR^+/+^ EMSCs exhibited deeper Alkaline phosphatase (ALP) staining and higher expression of odonto/osteogenic genes/proteins (e.g., dentin sialoprotein (DSPP) than p75NTR^+/+^ EMSCs. Moreover, p75NTR^+/+^ EMSCs exhibited higher nuclear P65 expression than p75NTR^−/−^EMSCs. Inhibition of NF-κB pathway with Bay11-7082 in p75NTR^+/+^EMSCs substantially decreased DSPP expression level. However, activation of NF-κB pathway with Bay11-7082 in p75NTR^−/−^EMSCs enhanced DSPP expression level. Thus, p75NTR possibly plays a paramount role in the proliferation and differentiation of EMSCs via NF-κB pathway.

## Graphical abstract

**Figure:**
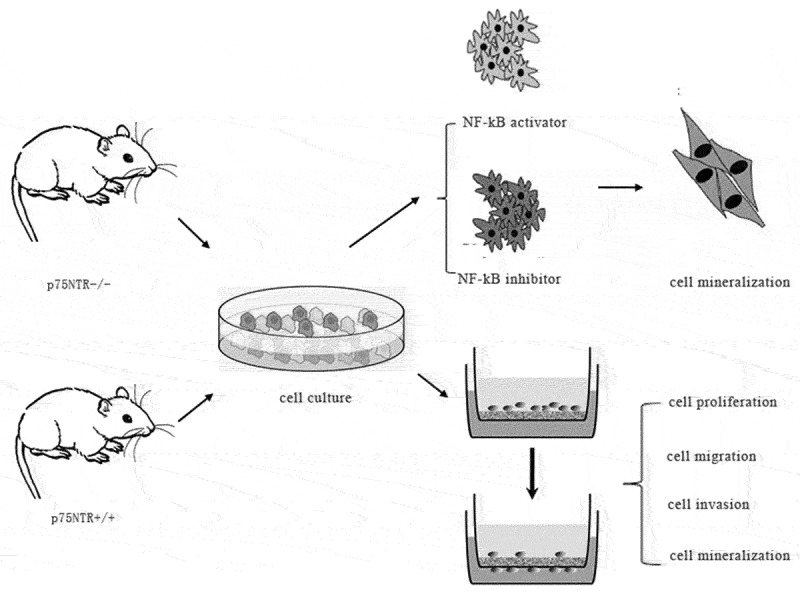

## Highlights

- p75NTR plays a paramount role in promoting odonto/osteogenic differentiation.
- p75NTR^+/+^EMSCs show more odonto/osteogenic ability than p75NTR^−/−^EMSCs.
- Inhibition of NF-κB may weaken the odonto/osteogenic ability of p75NTR^+/+^EMSCs.

## Introduction

The universal neurotrophin receptor p75NTR is a transmembrane glycoprotein belonging to the tumor necrosis factor (TNF) family [1]. Several studies have provided convincing evidence that p75NTR is involved in biological processes, such as cell proliferation [2], cell multi-differentiation [3,4], and apoptosis [5]. Notably, various recent studies have proven that p75NTR is essential for tooth morphogenesis and development [6,7]. It is also a reliable marker to purify cranial neural crest-derived EMSCs and develop a stem cell model that elucidates the mechanism of tooth restoration and regeneration [8]. From the perspective of tissue development, EMSCs are the cellular basis for the differentiation of odontogenesis which originates from the cranial neural crest subsequently differentiate into dental papilla, dentine, cementum and periodontal ligaments [9]. Multiple studies on p75NTR have focused more on the biological regulation of neural crest stem cells represented by EMSCs.

Studies that have investigated the signal transduction mechanism of p75NTR found that p75NTR is a central issue in the odonto/osteogenic differentiation of EMSCs [8]. Xing et al [10]. have demonstrated that p75NTR^+/+^EMSCs exhibited a higher in vivo and in vitro ability to odonto-differentiate than p75NTR^−/−^EMSCs. Furthermore, they validated that smad4 activation in p75NTR^+/+^EMSCs significantly upregulated the protein expression of DSPP and dentin matrix acidic phosphoprotein 1(DMP1). Zhao et al [11] have demonstrated that the combination of p75NTR and melanoma-associated antigen (MAGE) -d1 promotes tissue mineralization and osteogenesis. Thus, we hypothesized that p75NTR might be involved in the development of the mandible, which also originates from EMSCs.

NF-κB pathway is crucial for cell proliferation and survival, tooth morphogenesis and the modifications of MSCs [12]. Studies have reported that interferon gamma (IFN-γ) could regulate the behavior of human dental pulp stem cells via NF-κB and mitogen-activated protein kinase (MAPK) signaling pathways [13]. Notably, Yunnan Baiyao also induces the odonto/osteogenic differentiation of stem cells from apical papilla via NF-κB pathway [14]. Thus, these NF-κB regulatory mechanisms have similar effects as the p75NTR regulatory mechanisms. In addition, studies have reported an interaction between p75NTR and NF-κB; in the nervous system, the binding of neural growth factor and p75NTR enhances the survival of trigeminal neurons by activating NF-κB pathway [15]. Nonetheless, it is possible that both p75NTR and NF-κB have multiple interactions with each other. Bhakar et al [16] have proved that p75NTR does not directly activate NF-κB in fibroblasts; in fact, it indirectly enhances TNF -mediated NF-κB activation. These observations suggest that there may be a certain interaction between NF-κB and p75NTR. However, these studies do not fully elucidate the involvement of NF-κB pathway in the odonto/osteogenic differentiation of EMSCs.

In this study, we suggested that NF-κB pathway might promote the odonto/osteogenic differentiation of EMSCs. By investigating the function of p75NTR and its interaction with NF-κB in the odonto/osteogenic differentiation of EMSCs in vitro and in vivo, we decipher the role of NF-κB pathway in regulating the odonto/osteogenic differentiation of EMSCs. Thus, our study provides a theoretical basis for studying the role of NF-κB pathway in MSC modification and stem cell-based tooth regeneration.

## Materials and methods

### Ethics statement

All animal experiment protocols in this study were approved by the Medical Ethics Committee of the Wenzhou Medical University (NO.wydw2019-0224), and all experimental steps were performed according to ethical guidelines.

### Animal experiments

We raised p75NTR^−/−^ mice in the Wenzhou Medical University Animal Laboratory. The differences in body weights and appearances between p75NTR^−/−^ and p75NTR^+/+^ mice were compared. Additionally, we dissected the tail tissue for PCR analysis as previously described [17]. DNA extraction and PCR amplification were using one step mouse genotyping kit (Vazyme, Nanjing, China) according to the manufacturer’s protocol. The PCR products could be observed on a 3% agarose gel containing 5 mg/mL ethidium bromide.

### Cell isolation and culture

**Figure 1.**
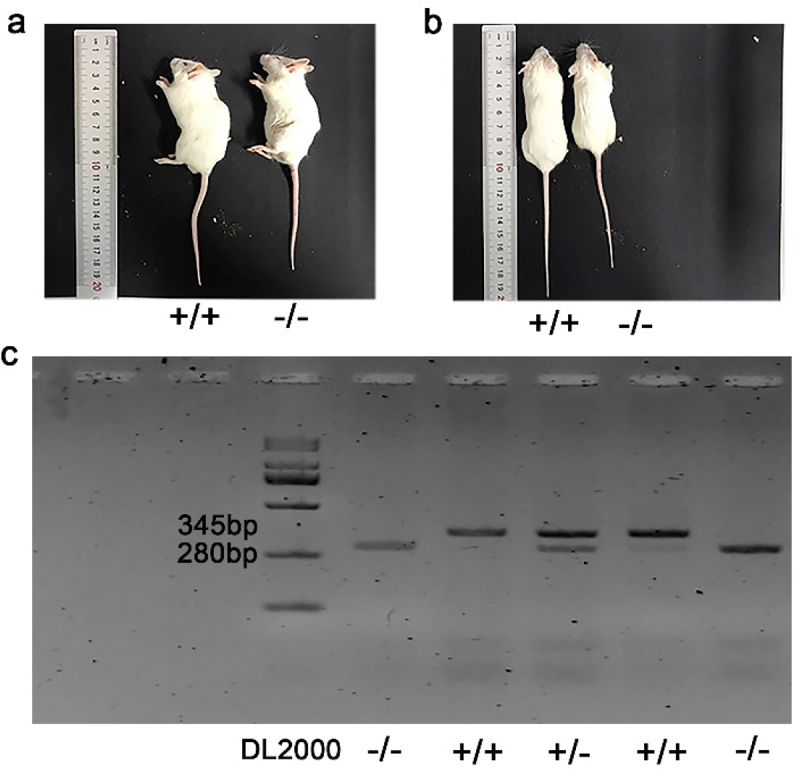
Cultivation and identification of EMSCs. (a, b) Embryos were separated from E16.5 p75NTR^±^ pregnant mice and the maxillofacial tissues were cut into pieces and cultured. (c-f) EMSCs showed a fibroblast-like morphology. (g) Mouse gene identification was shown in the figure. +/+ = p75NTR^+/+^, -/- = p75NTR^−/−^, ± = p75NTR^±^. Scale bar, 50 μm.

We obtained tissues from E16.5d p75NTR heterozygous pregnant mice (Figure 1a,b). Briefly, the embryonic mandibular processes were carefully isolated from each embryo (Figure 1c,d). According to previous study [18], primary EMSCs were extracted and cultured in dulbecco’s modified eagle medium: F 12 (DMEM/F12) (Gibco, USA), which including 10% fetal bovine serum (FBS) (Gibco, USA), 1% antibiotics (100 ug/ml penicillin and 100 µg/mL streptomycin). And finally cultured at 37°C in a humidified atmosphere comprising 5% CO_2_ (Figure 1e,f).

### Flow cytometry analysis

We harvested cells during their third passage and identified the cell markers SOX2, OCT4, CD29, CD45 by flow cytometry. In short, the cells(5X10^5^) were collected and fixed with polyoxymethylene for 30 minutes, and then incubated overnight at 4°C with the following antibodies:SOX2-APC(1:100,BioLegend,USA), OCT4-FITC, CD29-PE, CD45-FITC (1:100, BD Biosciences, USA. In each experiment, the corresponding mouse IgG isotype control antibodies combined with APC, FITC and PE were used as negative controls. Thereafter, p75NTR^+/+^ and p75NTR^−/−^EMSCs were analyzed using BD Accuri C6 Plus (BD Biosciences, USA).

### Scratch assay

We incubated p75NTR ^+/+^ and p75NTR^−/−^EMSCs (1X10^5^) onto a 12-well plate until the cells completely adhered to the plate. Scratches were made using a 200 μL tip on the plate; the width of the scratch was marked as the initial width. Phosphate buffer solution (PBS) was used to wash the cells in order to remove impurities and eliminate the edges of scratches. The width of the scratch post 24 h was considered as the final width. The formula for calculating the healing rate of scratches was as follows.

Scratch healing rate (%) = (0 h scratch width-24 h scratch width) /0 h scratch width × 100%.

### Transwell assay

We incubated p75NTR^+/+^ and p75NTR^−/−^EMSCs and p75NTR^+/+^ EMSCs- inhibitor containing 5 µmol/L Bay11-7082 (3 × 10^3^) in the upper part of a transwell chamber (Corning, USA) for 24 hours. Post incubation, the cells were washed with PBS, fixed with paraformaldehyde and stained with 0.1% crystal violet. Subsequently, we cleaned the upper chamber with a cotton swab. Cells that had migrated to the lower face of the membrane were observed under a microscope (Leica DM750, Germany) and cleaned with 3% acetic acid. Furthermore, the solution was collected and transferred to a 96-well plate, and the absorbance was measured at 490 nm using a microplate reader.

### CCK8 assay

For this assay, we seeded p75NTR^+/+^ and p75NTR^−/−^EMSCs and p75NTR^+/+^ EMSCs- inhibitor containing 5 µmol/L Bay11-7082 (2 × 10^3^) in 96-well plates. At 60% confluence, cells treated with 5 µmol/L NF-κB Bay11-7082 and cultured for 7 days continuously, CCK8 was performed according to the previous report [19].Subsequently, we added the cck8 solution (CCK8, Dojindo, Japan) to each well and incubated the cells for 2 hours at 37°C. Eventually, we measured the absorbance at 450 nm with a microplate reader.

### Preparation of dental epidermal cell conditioned medium

We prepared HAT-CM according to the method described previously [20,21]. In this regard, we first cultured the dental epithelial cell line HAT-7 in DMEM/F12 medium supplemented with FBS, 100 ug/ml penicillin and 100 µg/mL streptomycin. Once the cells reached 90% confluency, we centrifuged the supernatant at 2000 rpm for 30 minutes to remove the impurities and then filtered the supernatant through a 0.22 um filter. Following this, we added equal volumes of fresh DMEM/F12 to the filtrate and eventually stored at −80°C.

### Alkaline phosphatase and alizarin red staining

We plated p75NTR ^+/+^ and p75NTR^−/−^EMSCs and p75NTR^+/+^ EMSCs- inhibitor containing 5 µmol/L Bay11-7082 (5 × 10^4^) onto 24-well plates. Then the medium was subsequently replaced by HAT-CM. The induction medium was changed every 3 days. On days 7, the cells were washing three times with PBS, fixed in paraformaldehyde for 30 minutes. Following this, they were stained with an ALP kit (Beyotime, China) for 30 minutes. Thereafter, the fixed cells were washed twice with double-distilled water and observed under a microscope. ALP activity was performed by using an ALP activity kit [22] (Nanjing Jiancheng, China). Cells were plated onto 24-well plates. Then the medium was replaced by HAT-CM. And inhibitor (Bay11-7082) was added into p75NTR^+/+^ EMSCs. After 7 days of culture, ALP activity was measured. ALP activity of individual samples was normalized to total protein concentration.

After 2 weeks of induction, cells were fixed with 4% paraformaldehyde at room temperature for 20 min and stained with 0.2% alizarin red for 1 hour. For quantitative analysis, cells were destained with ethylpyridinium chloride for 30 minutes at room temperature. And we transferred it to a 96-well plate to measure the absorbance at 550 nm using a microplate reader.

### Immunohistochemistry

We separated the embryos from E16.5D mice and fixed them using an optimal cutting temperature compound (Sakura,USA) and 6um sections were made for immunostaining. We subsequently performed immunostaining using the following primary antibodies: NF-κB p65 Rabbit mAb (1:200, CST, USA), anti-CD271 (1:200, Invitrogen, USA). Specimens were combined with secondary antibody at room temperature and stained with DAPI (40,6-diamidino-2-phenylindole) (Abcam,USA). And then the sections were observed using a confocal microscope (Zeiss, LSM880, AxioObserver, Germany).

### Immunofluorescence

We fixed p75NTR^+/+^ and p75NTR^−/−^ EMSCs using 4% paraformaldehyde for 30 minutes at 4° and permeabilized in 0.5% triton X − 100 (Solarbio, Beijing, China). Following this, we blocked the cells using 1% BSA-PBS for 1 hour. The cells were subsequently incubated with NF-κB p65 rabbit primary mAb (1:200, CST, USA), following which they were incubated with anti-rabbit Alexa Fluor 555 conjugated secondary mAb (1:1000, Beyotime). The cell nuclei were counterstained with DAPI and cell images were obtained using a fluorescent microscope (Leica DMI8, Germany).

### Real-Time PCR

We cultured p75NTR^+/+^ and p75NTR^−/−^EMSCs and p75NTR^+/+^EMSCs-inhibitor containing 5 µmol/L Bay11-7082 and p75NTR^−/−^EMSCs-activator containing 10 ng/mL tumor necrosis factor-α (TNF-α) in the dental epidermal cell-conditioned medium. At 80% confluence, cells treated with 5 µmol/L NF-κB Bay11-7082. Real-time PCR was performed according to the method described previously [10]. We extracted total RNA from these cells using an RNA prep pure Cell/Bacteria Kit (TIANGEN, Beijing, China) as per the manufacturer’s protocol. The extracted RNA was reverse transcribed into cDNA using a HiScript® III RT Super Mix for qPCR kit (Vazyme, Nanjing, China). We then quantified the target genes by real-time RT-PCR using the ChamQ Universal SYBR qPCR Master Mix kit (Vazyme, Nanjing, China) and Real-Time PCR Detection System (Quantstudio5, USA). Relative gene expression levels were calculated by the comparative Ct (2-ΔΔCt) method. Primers used for real-time PCR were as follows: DSPP, 5'‐GGCTCCGAGTCAATACATGTA‐3' and 5'‐CTCCTTGGTGTCCATTGCTAT‐3'; runt-related transcription factor 2 (Runx2), 5'‐CTGCCACCTCTGACTTCTGC‐3' and 5'‐ GATGAAATGCCTGGGAACTG‐3'; Dmp1,5'‐ CAGAGGGACAGGCAAATAGTGAC‐3' and 5'‐ CATCGCCAAAGGTATCATCTCC‐3'; ALP, 5'‐ GGCTCTGCCGTTGTTTCTCT‐3'and 5'‐AAGGTGCTTTGGGAATCTGC‐3'; osteocalcin (OCN), 5'‐ CTTGGTGCACACCTAG CAGA‐3' and 5'‐ GCCGGAGTCTGTTCAC TACC‐3'; osterix (OSX), 5'‐ GCTGAGGAAGAAGCCCATTC‐3' and 5'‐ TTGGAGCAGAGCAGACAGGT‐3'; glyceraldehyde- 3 – phosphate dehydrogenase (GAPDH), 5'‐ ACAGCAACAGGGTGGTGGAC‐3' and 5'‐ TTTGAGGGTGCAGCGAACTT‐3'.

### Western blot

We harvested p75NTR^+/+^ and p75NTR^−/−^EMSCs and p75NTR^+/+^ EMSCs-inhibitor containing 5 µmol/L Bay11-7082 and p75NTR^−/−^EMSCs-activator containing 10 ng/mL TNF-α after 7 days. Subsequently, we dissolved the cells in RIPA lysis buffer (Beyotime, Beijing, China) supplemented with 1 mM phenylmethylsulfonyl fluoride (Beyotime, Beijing, China) on ice for 20 minutes. We determined the protein concentrations in the cells using the BCA Protein Assay Kit) (Beyotime, Beijing, China). Thereafter, we separated these proteins (20 μg/lane) 10% sodium dodecyl sulfate polyacrylamide gel electrophoresis. The separated proteins were subsequently transferred onto Sequi-Blot™ PVDF and the membranes (BIO-RAD, USA) were blocked with 5% BSA. The primary antibodies used in this experiment were as follows: rabbit anti- polyclonal RUNX2 (1: 1000, Abcam,USA), rabbit anti- polyclonal DSPP (1: 1000, Absin,USA), rabbit anti- polyclonal P- NF-κB (1: 1000,CST,USA), rabbit anti- polyclonal NF-κB (1:1000, CST,USA), and mouse anti- polyclonal β-ACTIN (1: 8000, CST,USA), IRDye® 800CW Goat anti-Rabbit(1:5000,LI-COR,USA) and IRDye® 680RD Goat anti-Mouse(1:5000,LI-COR,USA).The membranes were visualized and scanned by Odyssey Clx(LI-COR,USA). The grayscale analysis was performed using Image J.

### Statistical analysis

All results are presented as mean ±standard deviation (SD). Statistical analysis was performed using GraphPad Prism software. Comparisons between groups were assessed by paired student t-test. Multiple groups were analyzed by two-way analysis of variance (ANOVA). Value of P < 0.05 was considered as statistically significant.

## Results

In this study, we aimed to explore the function of p75NTR and its interaction with NF-κB in the odonto/osteogenic differentiation of EMSCs. In vivo, we found that p75NTR and NF-κB were highly expressed in p75NTR^+/+^ mice. In vitro, we successfully isolated EMSCs from p75NTR^±^pregnant mouse embryos, and confirmed that p75NTR^+/+^EMSCs has stronger odonto/osteogenic differentiation than p75NTR^−/−^EMSCs and then confirmed that NF-κB has a positive regulation on the odontogenic/osteogenic differentiation of EMSCs.

### Genotype identification of p75NTR-knockout mice

**Figure 2.**
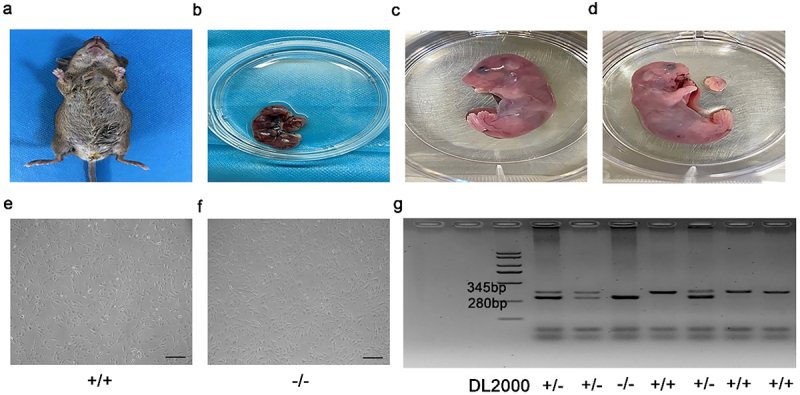
Gene identification and appearance comparison of p75NTR^+/+^ and p75NTR^−/−^ mice. (a, b) Visual observation of p75NTR^+/+^ and p75NTR^−/−^ mice. (c) The band detected at 280bp was p75NTR^±^mice, at 345bp was p75NTR^+/+^ mice, and two bands appear simultaneously were p75NTR^±^mice. +/+ = p75NTR^+/+^, -/- = p75NTR^−/−^, ± = p75NTR^+/^.

The mouse genotypes are presented in Figure 2c. The littermates that were detected with two bands at 280 bp and 345 bp were identified as heterozygous mice. On the other hand, those detected with only one band at either 280 bp or 345 bp were identified as p75NTR^−/−^mice or p75NTR^+/+^mice, respectively. Significant differences in body size were observed when the littermates grew to 8 weeks of age. Notably, p75NTR^+/+^mice(length 9.833 ± 0.7638 cm; weight 29.78 ± 1.418 g) were bigger than p75NTR^−/−^mice (length 8.467 ± 0.8737 cm; weight 24.92 ± 1.268 g) (Figure 2a,b).

### Identification of EMSCs

**Figure 3.**
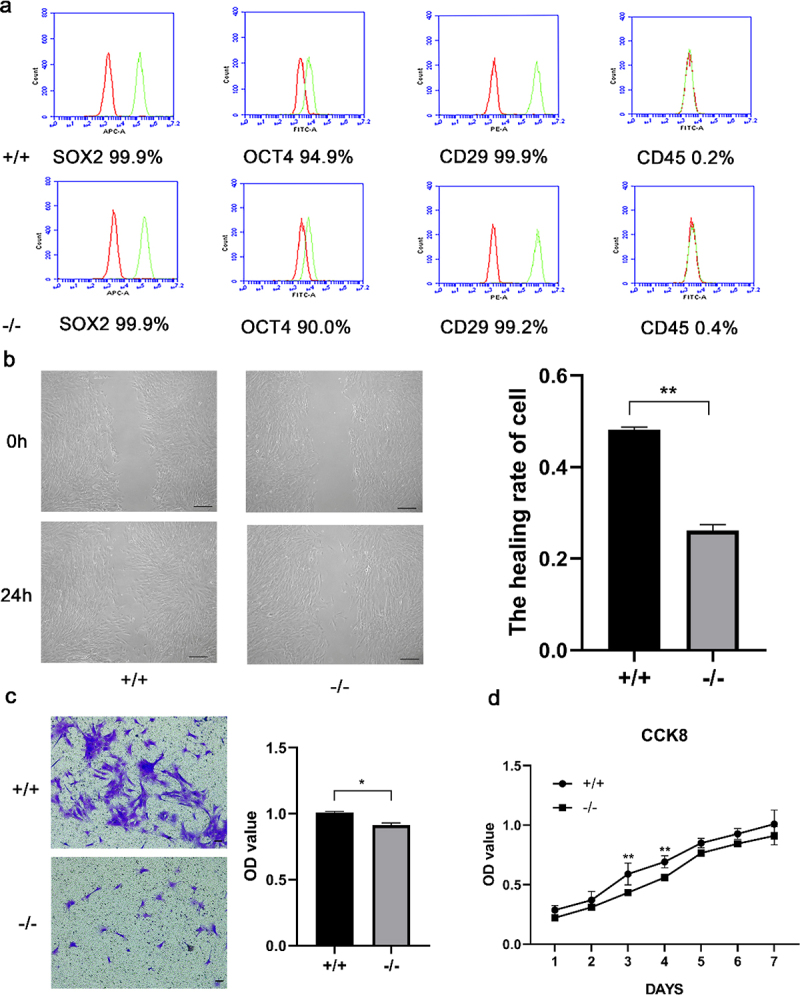
Characterization of p75NTR^+/+^ and p75NTR^−/−^ EMSCs. (a) Flow cytometry analysis showed that EMSCs was positively expressed in MSC markers SOX2, OCT4 and CD29, but negatively expressed in hematopoietic marker CD45. (b) The migration ability of p75NTR^+/+^ and p75NTR^−/−^ EMSCs was performed by wound healing assay and quantitative measurement of the healing rate of EMSCs. (c) Images of the transwell assay and quantitative measurement of the migration rate of EMSCs. (d) Growth curves of p75NTR^+/+^ and p75NTR^−/−^ EMSCs. The data are presented as mean ± SD, n = 3, *P < 0.05, **P < .01, ***P < .001. Scale bar, 50 μm.

The mouse embryo genotypes are presented in Figure 1g. We observed that both p75NTR^+/+^ and p75NTR^−/−^EMSCs showed fibroblast-like morphology. While EMSCs had a high expression of the MSC markers OCT4, SOX2, CD29, they had a decreased expression of the hematopoietic marker CD45, as revealed by our flow cytometry data (Figure 3a). These results demonstrated that p75NTR^+/+^ and p75NTR^−/−^EMSCs had characteristics similar to that of MSCs, providing a theoretical foundation to the odonto/osteogenic differentiation.

### Migration and proliferation of p75NTR^+/+^ and p75NTR^−/−^ EMSCs

Next, we investigated the migration of p75NTR^+/+^ and p75NTR^−/−^ EMSCs by scratch and transwell assays. In the scratch assay, the migration ability of p75NTR^+/+^EMSCs significantly stronger than p75NTR^−/−^EMSCs. Moreover, a quantitative analysis indicated that approximately 74% of the wounded gap in p75NTR^−/−^EMSCs remained unclosed compared with <52% gap open in p75NTR^+/+^ EMSCs (Figure 3b). Similarly, our transwell assay data also indicated that p75NTR^+/+^ EMSCs had a higher migrative ability than p75NTR^−/−^ EMSCs (Figure 3c). We discovered that the proliferation of p75NTR^+/+^ and p75NTR^−/−^ EMSCs by CCK8. We also evaluated the proliferation was rapid on day 3 and gradually decreased on day 5 (Figure 3d).

### The odonto/osteogenic ability of p75NTR^+/+^ and p75NTR^−/−^ EMSCs

**Figure 4.**
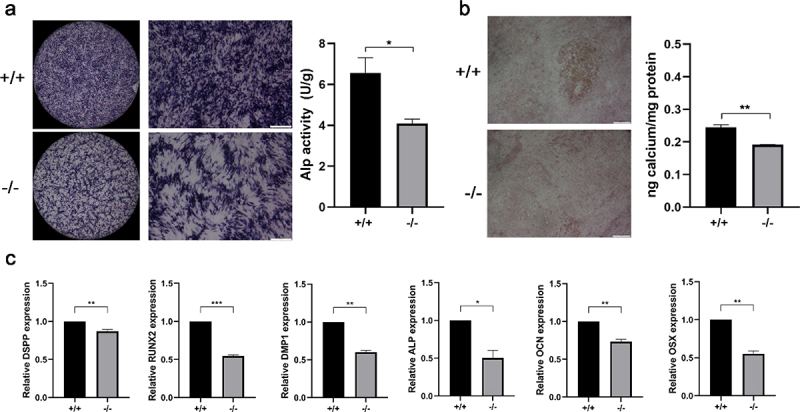
Mineralization assay. (a) Under induction with dental epithelium conditioned medium for 7 days, p75NTR^+/+^ and p75NTR^−/−^EMSCs were used for ALP staining assay. ALP activities of p75NTR^+/+^EMSCs was significantly higher compared with p75NTR^−/−^ EMSCs. (b) p75NTR^+/+^ and p75NTR^−/−^ EMSCs were used for Alizarin red staining. Calcium quantification illustrated the weaker calcium deposition in p75NTR^−/−^ EMSCs compared with p75NTR^+/+^EMSCs. (c) The mRNA expression of DSPP, Runx2, DMP1, ALP, OCN and OSX were examined by RT-PCR. The data are presented as mean ± SD, n = 3, *P < 0.05, **P < .01, ***P < .001. Scale bar, 100 μm.

ALP staining assay and alizarin red staining were used to measure the odonto/osteogenic differentiation. The results revealed that the color of ALP staining in p75NTR^+/+^ EMSCs was deeper compared with that in p75NTR^−/−^ EMSCs. And ALP activity in p75NTR^+/+^ EMSCs was significantly higher compared with p75NTR^−/−^ EMSCs (Figure 4a). On day 14, the calcified nodules of p75NTR^+/+^EMSCs were significantly higher than p75NTR^−/−^EMSCs. Furthermore, quantitative calcium measurements also showed more calcifications in p75NTR^+/+^EMSCs than in p75NTR^−/−^ EMSCs (Figure 4b). Moreover, we measured the gene expression of odonto/osteogenic markers by Real-time PCR and found that the expressions of DSPP, Runx2, DMP1, ALP, OCN and OSX were higher in p75NTR^+/+^ EMSCs than those in p75NTR^−/−^EMSCs (Figure 4c).

### The expression of p75NTR and NF-κB in p75NTR^+/+^and p75NTR^−/−^ mice

**Figure 5.**
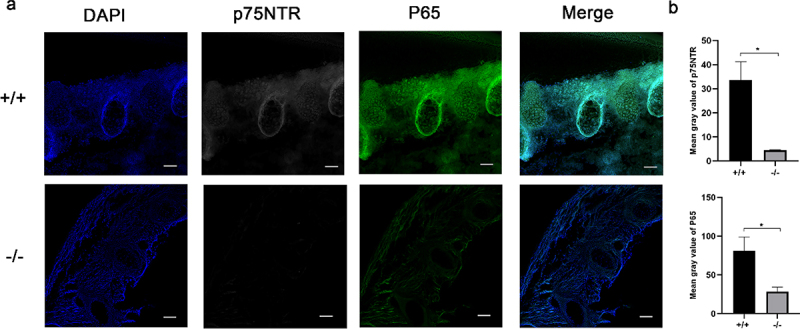
Immunohistochemistry exhibited similar expression patterns for p75NTR and NF-κB. (a) The confocal laser scanning microscopy results revealed that p75NTR and P65 were strongly expressed in the spine of the trunk of p75NTR^+/+^mice, but weakly expressed in p75NTR^−/−^mice. (b) Quantifications of immunostaining for p75NTR and P65. The data are presented as mean ± SD, n = 3, *P < 0.05. Scale bar, 50 μm.

We selected the developmental mineralization model of the mouse embryonic spine to decipher the expression patterns of p75NTR and NF-κB in vivo. The epiphyseal ring was the germinal center of bone tissue and the osteoblast colony. The immunostaining of p75NTR protein is highly expressed in the spine of the trunk of p75NTR^+/+^ mice, but weaker in p75NTR^−/−^ mice (Figure 5a,b). Notably, NF-κB exhibited an identical expression trend in the mice spine.

### Effects of NF-κB pathway on the proliferation and invasion of EMSCs

**Figure 6.**
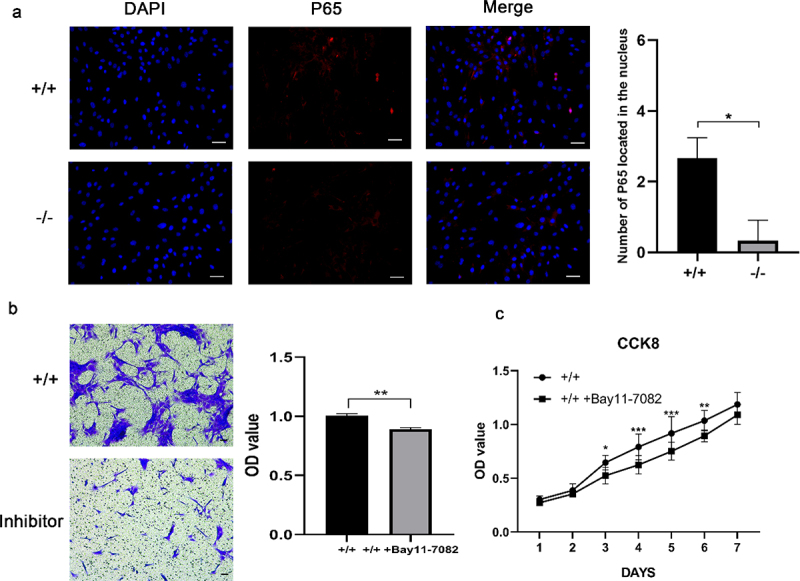
The proliferation and migration of EMSCs were slowed down by inhibition of NF-κB. (a) Immunofluorescence staining of P65 in p75NTR^+/+^ and p75NTR^−/−^EMSCs and the number of P65 in the nucleus. Almost no nucleic accumulation of P65 was demonstrated in p75NTR^−/−^EMSCs. (b) Transwell was used for analyzing the invasion of p75NTR+/+ and p75NTR^+/+^EMSCs with inhibitor. (c) CCK8 assay was used for detecting the proliferation of p75NTR^+/+^ and p75NTR^+/+^EMSCs with inhibitor. The data are presented as mean ± SD, n = 3, *P < 0.05, **P < .01, ***P < 001. Scale bar, 50 μm.

Immunofluorescence staining revealed that the amount of NF-κB located in the nucleus increased in p75NTR^+/+^EMSCs than p75NTR^−/−^EMSCs (Figure 6a). Moreover, in contrast to p75NTR^+/+^EMSCs, inhibition of NF-κB pathway impaired the migration capacity of p75NTR^+/+^EMSCs (Figure 6b). In addition, as shown in Figure 6c, p75NTR^+/+^ and p75NTR^+/+^EMSCs-inhibitor exhibited no significant difference in cell growth within 48 hours followed by rapid cell proliferation of p75NTR^+/+^ EMSCs. These findings indicated that NF-κB pathway facilitated the proliferation and invasion of EMSCs.

### Effects of NF-κB pathway on the odonto/osteogenic differentiation of EMSCs

**Figure 7.**
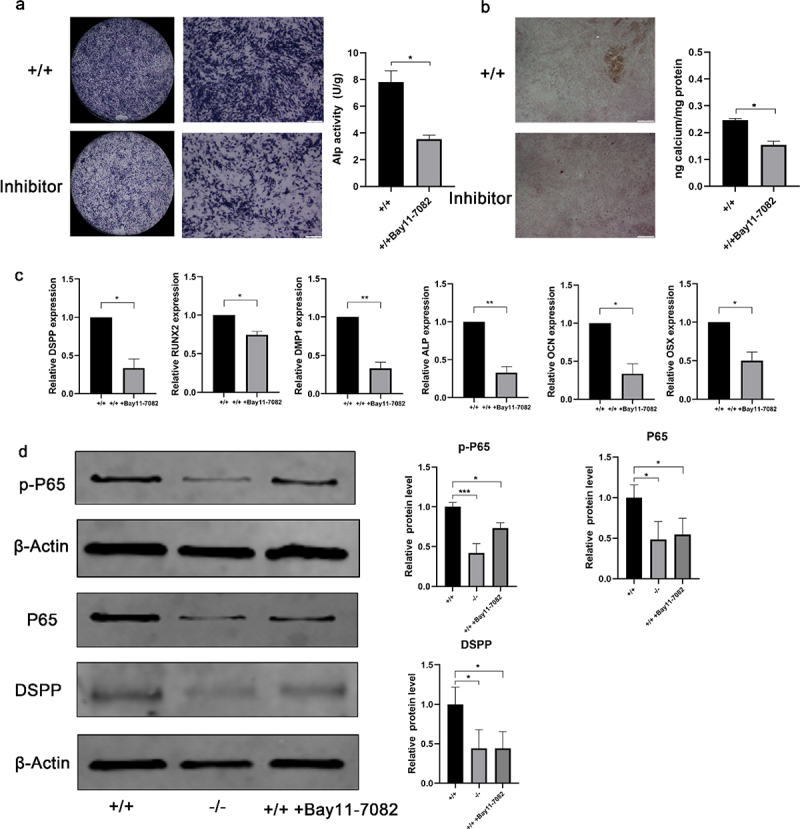
Odonto/osteogenic differentiation in NF-κB-inhibited EMSCs. (a) Under induction with dental epithelium conditioned medium for 7 days, ALP staining was used to detect the potential of odonto/osteogenic differentiation in p75NTR^+/+^EMSCs group and p75NTR^+/+^EMSCs-inhibitor group. ALP activities of p75NTR^+/+^EMSCs-inhibitor was significantly lower compared with p75NTR^+/+^EMSCs. (b) Alizarin red staining was used to detect the potential of odonto/osteogenic differentiation in p75NTR^+/+^EMSCs group and p75NTR^+/+^EMSCs-inhibitor group. Calcium quantification illustrated the higher calcium deposition in p75NTR^+/+^EMSCs compared with p75NTR^+/+^EMSCs-inhibitor group. (c) RT-PCR for the detection of DSPP, Runx2, DMP1, ALP, OCN and OSX of EMSCs, respectively, p75NTR^+/+^ EMSCs, p75NTR^−/−^ EMSCs, and p75NTR^+/+^ EMSCs + inhibitor groups at day 7. (d) After 7 days of induction, the expression levels of P65, p-P65 and DSPP were detected by western blot, β-Actin used as the reference gene. Semiquantitative analysis demonstrated that the expression odonto /osteogenic marker (DSPP) was significantly downregulated in NF-κB pathway-inhibited EMSCs than those in control group at day 7. The data are presented as mean ± SD, n = 3, *P < 0.05, **P < .01, ***P < .001. Scale bar, 100 μm.

ALP staining of inhibition-treated p75NTR^+/+^ EMSCs in dental epidermal cell conditioned medium was reduced at day 7, as compared with untreated groups. And ALP activity was significantly reduced in p75NTR^+/+^EMSCs-inhibitor compared with p75NTR^+/+^EMSCs (Figure 7a). Furthermore, ARS staining showed decreased intracellular calcium deposition after the addition of the inhibitor (Figure 7b). The expression of related odonto/osteogenic genes (DSPP, Runx2, DMP1, ALP, OCN and OSX) were detected by real-time PCR assays. In inhibitor-treated p75NTR^+/+^EMSCs, expression of odonto/osteogenic genes was significantly downregulated especially DMP1 and ALP (Figure 7c). Conversely, in activator-treated p75NTR^−/−^EMSCs, expression of odonto/osteogenic genes was significantly upregulated (Supplementary Fig. 8a). DSPP odontization/mineralization-related marker, was significantly downregulated when NF-κB was suppressed by adding inhibitor after 7 days coculture (Figure 7d). In addition, when NF-κB activator is induced in p75NTR^−/−^ cells, the odontoblast/osteoblast differentiation ability of p75NTR^−/−^ cells was enhanced (Supplementary Fig. 8b), which was similar to the real-time PCR results.

## Discussion

Tooth development is a long-term, continuous and complex process including a variety of complex epithelial mesenchymal interactions [23,24]. During this process, EMSCs migrate from the cranial neural crest to the branchial arches and differentiate to form dental tissues, such as dentin, pulp, cementum, periodontal ligament and alveolar bone [25]. However, the mechanism governing this cell differentiation has not been well studied. Importantly, studies showed that p75NTR to be a specific surface marker of neural crest-derived stem cells [26]. Thus, it can be used to purify neural crest-derived EMSCs and to regulate tooth morphogenesis, osteogenic differentiation and tissue mineralization [11,27,28]. In this study, p75NTR^+/+^EMSCs show a higher proliferation and odonto/osteogenic differentiation than p75NTR^−/−^ EMSCs. Our findings revealed that p75NTR possibly up-regulates the odonto/osteogenic differentiation of stem cells and the formation of hard tissue.

However, the role of p75NTR in promoting the mineralization of EMSCs is controversial. For instance, Yoshikazu Mikami et al [29]. have demonstrated p75NTR signaling is related to trk tyrosine kinase (TRKA) receptor and promotes the proliferation and differentiation of pre-osteoblast. In contrast, a study demonstrated that p75NTR inhibited the osteogenic differentiation in C3H10T cells [30].In this study, we isolated EMSCs from the first branchial arch of mouse embryos. Both p75NTR^+/+^ and p75NTR^−/−^ EMSCs exhibited fibroblast-like morphology and were characterized as MSCs. Additionally, expression levels of the MSC markers, which included OCT4, SOX2, CD29 and CD45, hardly differed among EMSCs and MSCs. Thus, we validated that EMSCs originated from the cranial neural crest. However, p75NTR^+/+^ EMSCs showed higher proliferation than p75NTR^−/−^ EMSCs. Besides, deeper ALP staining was seen in p75NTR^+/+^ EMSCs, and the mineralization-related genes DSPP, RUNX2, DMP1, ALP, OSX and OCN were also expressed at high levels. These results confirmed that p75NTR^+/+^ EMSCs have unparalleled advantages in odontogenesis and mineralization. Furthermore, we validated that the change in p75NTR does not affect the stem cell characteristics, as it still promotes odonto/osteogenic differentiation.

Since NF-κB is a pleiotropic transcription factor, it regulates several biological phenomena, such as cell growth, apoptosis and differentiation [31–33]. In the nervous system, inhibiting the NF-κB pathway can reduce the inflammatory response, thereby ameliorating spinal cord injury in rats [34]. Previous research has showed that NF-κB pathway plays different roles during stem cell differentiation. For example, Chang et al [35]. have discovered that NF-κB pathway could inhibit the osteogenic differentiation of mesenchymal stem cells. Conversely, Wang et al [36] have proved that activation of NF-κB pathway could enhance odonto/osteogenic ability of stem cells from apical papilla. Therefore, exploring the role of NF-κB pathway on differentiation in different cells is of great significance. In our in vivo experiments, we found that NF-κB and p75NTR were highly expressed in the germinal center of the developing spine of a mouse embryo; this expression differed in other tissues. Additionally, they were specifically expressed in the cell cytoplasm around the spine, forming a ring structure; however, the adjacent soft tissue of the intervertebral disc had no obvious expression. This reveals the possible involvement of this gene in the mineralization of bone tissue. Meanwhile, we found that the developmental changes in the spine indirectly determined the body length of the mice. This experiment was also confirmed in the initial study of body weight and length of mutant mice. On the basis of animal experiments, we further revealed the role of NF-κB in EMSCs at the cellular level. The number of NF-κB located in the nucleus of p75NTR^−/−^EMSCs was significantly less than that of p75NTR^+/+^EMSCs. In addition, we found that there are significant differences in the cell proliferation and migration abilities between the two types of stem cells. NF-κB – inhibitor-treated EMSCs shown a more ineffective migration in transwell, and lower proliferation index in CCK8 than NF-κB – inhibitor-untreated EMSCs. Furthermore, the number of migrated cells in NF-κB – inhibitor-treated EMSCs post 24 h of treatment were less than untreated cells. These findings indicated that NF-κB pathway is likely to be a key for the cell multiplication and motility.

In this study, with the blockage of NF-κB pathway, inhibitor-treated cells showed lower ALP activity, and the odonto/osteogenic genes DSPP, DMP1, Runx2, ALP, OCN and OSX were also expressed at low levels. Conversely, activator-treated cells showed higher ALP activity, and the odonto/osteogenic genes were also expressed at high levels. These findings were consistent with our observation p75NTR^+/+^EMSCs show higher expression of NF-κB than that of p75NTR^−/−^EMSCs. Furthermore, inhibition of NF-κB pathway leads to a decrease in the protein of odonto/osteogenic markers, which reveals that NF-κB pathway is associated with the odonto/osteogenic differentiation of EMSCs. Therefore, we believe that inhibition of NF-κB pathway may reduce the ability of EMSCs to undergo odonto/osteogenic differentiation.

## Conclusion

In summary, data accumulated here suggested that p75NTR^+/+^ EMSCs exhibited more odonto/osteogenic differentiation than p75NTR^−/−^ EMSCs. Constantly Inhibiting NF-κB could diminish the odonto/osteogenic differentiation of EMSCs. Thus, our findings may enrich the biological functional characteristics of EMSCs and help clarify the functions in tooth development and regeneration.

## Supplementary Material

Supplemental Material
