# Supplementary material for: P75 neurotrophin receptor positively regulates the odontogenic/osteogenic differentiation of ectomesenchymal stem cells via nuclear factor kappa-B signaling pathway
Source: Bioengineered. 2022 Apr 29;13(4):11201–13. doi: 10.1080/21655979.2022.2063495 (PMC9208484; doi:10.1080/21655979.2022.2063495)
Supplement: Supplemental Material [file KBIE_A_2063495_SM4325.docx]

**Figure legends**


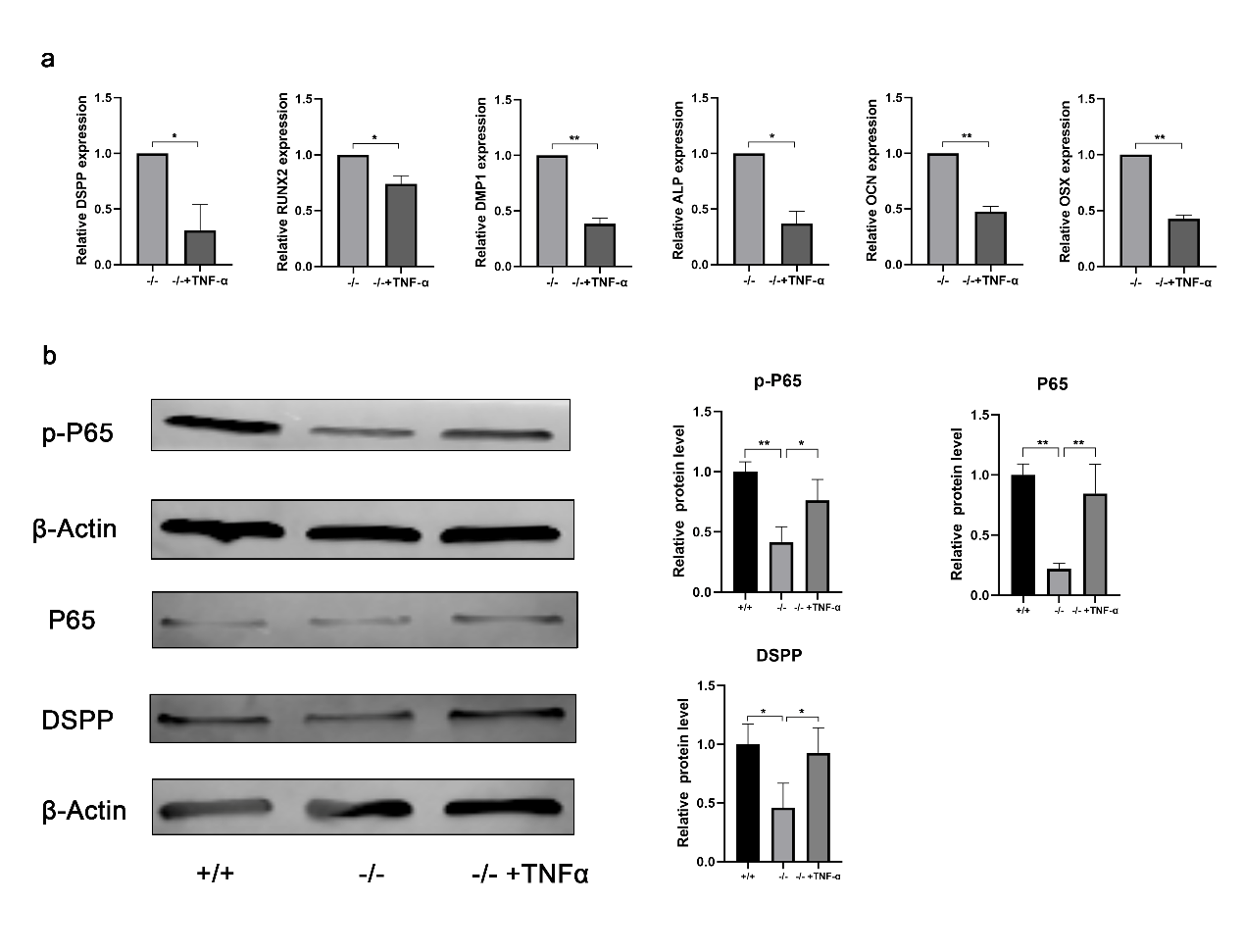


**Fig.7** Odonto/osteogenic differentiation in NF-κB-activated EMSCs. (a) RT-PCR was used to detect DSPP, Runx2, DMP1, ALP, OCN, and OSX in EMSCs, p75NTR^+/+^ EMSCs, p75NTR^-/-^ EMSCs, and p75NTR^+/+^ EMSCs+ activator groups. (b) Western blot was used to detect the expression levels of P65, p-P65 and DSPP, with β-Actin as the internal reference gene. Semiquantitative analysis showed that the expression of odonto /osteogenic markers (DSPP) was significantly higher in NF-κB pathway-activated EMSCs than in the control group at day 7. The data are presented as mean ± SD, n = 3, *P <0.05, **P < .01.
